# Implementation of novel boolean logic gates for IMPLICATION and XOR functions using riboregulators

**DOI:** 10.1080/21655979.2021.2020493

**Published:** 2022-01-05

**Authors:** Chaoxin Chen, Qi Wu, Qingying Ke, Ting Wang, Yifan Zhang, Feiwen Wei, Xiaolong Wang, Guanglei Liu

**Affiliations:** a The iGEM Laboratory of OUC-China, College of Marine Life Sciences, Ocean University of China, Qingdao, China; b Laboratory for Marine Biology and Biotechnology, Qingdao National Laboratory for Marine Science and Technology, Qingdao, Shandong, China

**Keywords:** Biological boolean logic gates, implication (IMP), XOR, RNA regulatory elements, riboregulator, synthetic RNA switches

## Abstract

To date, several different types of synthetic genetic switches, including riboregulators, riboswitches, and toehold switches, have been developed to construct AND, OR, NOT, NAND, NOR, and NOT IMPLICATION (NIMP) gates. The logic gate can integrate multiple input signals following a set of algorithms and generate a response only if strictly defined conditions are met. However, there are still some logic gates that have not been implemented but are necessary to build complex genetic circuits. Here, based on the toehold switches and three-way-junction (3WJ) repressors, we designed two novel biological Boolean logic gates of IMPLICATION (IMP) and XOR. Subsequently, the outputs of these two logic gates were characterized by fluorescence analysis, indicating that they can achieve the truth tables of logical gates. Furthermore, the fluorescence intensity under the logical TRUE condition was significantly higher than under the logical FALSE condition, suggesting the high dynamic range of the ON/OFF ratios. Because of the programmability of synthetic RNA switches, the constructed RNA logic gates could serve as elementary units to build a versatile and powerful platform for translational regulation and RNA-based biological computation.

## 1. Introduction

One of the goals of synthetic biology is to create engineered biological modules with various functions. Biological circuits can be implemented in living organisms, thereby regulating the movement of life and performing different tasks. DNA, RNA, and proteins all have the potential to be assembled into circuits for the design of programmable modules [1–3]. In addition, biocomputing systems can be developed with multiple stimulus-responsive film electrodes and bioelectrocatalysis based Boolean logic gates [4–6]. However, the standardization of such modules is still facing many problems, including cross-talk, noise, and mutations [7]. Consequently, the new classes of biological modules that offer a wide dynamic range, low system cross-talk, and design flexibility, are necessary to transform cells into living machines that can be precisely tuned by human beings [8]. Furthermore, they represent an enabling step toward fully achieving a biological computation system with inputs and outputs to carry out specific functions [7].

Among the regulatory elements, synthetic RNA switches are quite favorable in many respects due to their advantages and unique features. Since RNA is an intermediate between DNA and protein, RNA switches can respond to different regulatory elements. Furthermore, a wide assortment of RNA-based parts operating at the transcriptional and post-transcriptional levels are known to exist in nature [3]. Because the regulatory function of RNA relies on the Watson-Crick base pairing rule, the RNA secondary structures could be designed and predicted *in silico* [9]. In addition, RNA can be translated into output protein directly, which is more flexible and time-saving compared to DNA elements [1,10].

So far, researchers have developed several different types of synthetic genetic switches, including riboregulators, riboswitches, and toehold switches [9,11,12]. Genetic circuits combine a series of synthetic switches into networks that can perceive a signal, process the information, and generate an output, normally triggering gene expression and expression of a reporter to monitor a process or activation of a metabolic pathway [13]. Relatively, toehold switches have less cross-talk and higher ON/OFF ratios than other regulatory elements, making them be candidate tools for constructing complex circuits [9]. Another RNA repressor with a similar structure, called three-way-junction (3WJ) repressor, has also demonstrated good fold reduction [14]. Both switches employ toehold mediated interaction and a stem-loop structure, with a ribosome binding site (RBS) positioned in the loop, and the start codon is kept in a bulge. The binding of trigger RNA with toehold switch can disassemble the stem-loop structure, thereby initiating the translation of the output gene. However, the 3WJ repressor has an unstable structure, so the binding of its trigger RNA can only stabilize the structure, repressing downstream translation [9,14]. In recent years, toehold switches and 3WJ repressors have been applied in many studies [15,16], including constructing biological Boolean logic gates [17,18]. The logic gates can integrate multiple input signals following a set of algorithms and generate a response only if strictly defined conditions are met [13].In terms of logic gates based on toehold switches and 3WJ repressors, trigger RNAs are used as the inputs, and fluorescence proteins were taken as the output to visualize the activation or repression of the reporter gene. Currently, AND, OR, NOT, NAND, NOR, NOT IMPLICATION (NIMP) gates have been achieved. Thus, these logic gates can be applied to build complex gene circuits and even be used as basic units of biocomputers [14,17,18]. However, the logic gates of RNA-only IMPLICATION (IMP), XOR, and XNOR, which are also necessary to build biocomputers, have not been constructed [14,17].

It has been reported that the logic function can be implemented by toehold switches or 3WJ repressors [14,17]. So, we hypothesize that novel logic gates can be implemented by connecting different types of RNA switches or by using trigger pairs whose ends can be combined. In this study, to construct RNA-only logic gates of IMP and XOR based on RNA elements, the sequences and structures of numerous available RNA switches were analyzed to select suitable elements. The constructed IMP and XOR were further evaluated by the analysis of truth table, dynamic range, cross-talk, logic computing power, indicating these logic gates have the potential to regulate gene expression precisely and serve as a reliable diagnostic tool for specific RNAs detection and RNA-based biological computation.

## 2. Materials and methods

### 2.1 Plasmids construction

All DNA oligonucleotides and plasmids construction were from GENEWIZ. pUC19 plasmid was used as the vector for cloning the trigger RNA, and a pACYC184 plasmid was used for the switch RNA. Segments of the trigger were inserted into pUC19 by the site *Kpn* I (5ʹ) and site *Aat* II (3ʹ). Segments of the switch were inserted into pACYC184 by the site *SnaB* I (5ʹ) and the site *Hind* III (3ʹ). Plasmids were extracted using TIAN prep Mini Plasmid Kit II (TIANGEN Code No. DP106-02).

The constructed plasmids were transformed into the *E. coli* strain to verify the two-plasmid system. Upstream and downstream primers (Table 1) were designed and used for colony Polymerase Chain Reaction (PCR). All trigger segments had the same sequences at 5ʹ end and 3ʹ end and were inserted into pUC19 by the same sites. As a result, primers *F2-pTet+GGG* and *R2-Contain Aat* II were applicable for all trigger RNA. Similarly, primers *F1-T7 General purpose primer* and *R1-the frame of GFP* were applicable for all switch RNA. The primers and sequences used for experiments are listed in Table 1.

**Table 1.** Primers used in this study

Name	Sequence	Function
F1-T7 General purpose primer	TAATACGACTCACTATAGGG	verifying switch plasmids
R1-the frame of GFP	TTTTCGTCGTTTGCTGCAGG	verifying switch plasmids
F2-pTet+GGG	TTTCACACATCAACGGG	verifying trigger plasmids
R2-Contain *Aat* II	AAAAGTGCCACCTGACGTCA	verifying trigger plasmids

### 2.2 Strains and growth conditions

*Escherichia coli* strains BL21 Star DE3 (*F− ompT hsdSB (rB− mB−) gal dcm rne131* (DE3); Shanghai Yuanye Bio-Technology Co., Ltd) and DH5α (*F- φ80 lac ZΔM15 Δ(lacZYA-arg F) U169 endA1 recA1 hsdR17(rk-,mk+) supE44λ- thi −1 gyrA96 relA1 phoA*; GENEWIZ) were used in this study. DH5α was for amplification, and BL21 Star DE3 was for verification. The *E. coli* competent cells were prepared and transformed using standard molecular biology techniques. After *E. coli* were cultured in Luria-Bertani (LB) agar plates for 24 h, the robust colonies were picked into 250 mL conical flasks containing 50 mL LB broth. The cells were shake-flask cultured at 37°C and 200 rpm for 10 h with 50 μg·mL^−1^ ampicillin or/and 34 μg·mL^−1^ chloramphenicol. All the strains used in this study are listed in Table 2.

**Table 2.** Strains used in this study

Strains	Plasmids	Function
PROM1	pUC19-IPtet-tetR	Screening the promoter with low leakage and high dynamic range
PROM2	pUC19-pTet-tetR	Screening the promoter with low leakage and high dynamic range
PROM3	pUC19-pT7	Screening the promoter with low leakage and high dynamic range
PROM4	pUC19-pLacI-LacI	Screening the promoter with low leakage and high dynamic range
PROM5	pUC19-pBAD-araC	Screening the promoter with low leakage and high dynamic range
PROM6	pUC19-pRhaB-rhaS-rhaR	Screening the promoter with low leakage and high dynamic range
PROM7	pUC19-pluxPR_4G12T-luxR	Screening the promoter with low leakage and high dynamic range
PROM8	pUC19-pluxPR-luxR	Screening the promoter with low leakage and high dynamic range
T1S1	pUC19-pTet-trigger1-tetR; pACYC184-pT7-switch1-LacI	Verifying the orthogonality
T1S2	pUC19-pTet-trigger1-tetR; pACYC184-pT7-switch2-LacI	Verifying the orthogonality
T1S3	pUC19-pTet-trigger1-tetR; pACYC184-pT7-switch3-LacI	Verifying the orthogonality
T1S4	pUC19-pTet-trigger1-tetR; pACYC184-pT7-switch4-LacI	Verifying the orthogonality
T2S1	pUC19-pTet-trigger2-tetR; pACYC184-pT7-switch1-LacI	Verifying the orthogonality
T2S2	pUC19-pTet-trigger2-tetR; pACYC184-pT7-switch2-LacI	Verifying the orthogonality
T2S3	pUC19-pTet-trigger2-tetR; pACYC184-pT7-switch3-LacI	Verifying the orthogonality
T2S4	pUC19-pTet-trigger2-tetR; pACYC184-pT7-switch4-LacI	Verifying the orthogonality
T3S1	pUC19-pTet-trigger3-tetR; pACYC184-pT7-switch1-LacI	Verifying the orthogonality
T3S2	pUC19-pTet-trigger3-tetR; pACYC184-pT7-switch2-LacI	Verifying the orthogonality
T3S3	pUC19-pTet-trigger3-tetR; pACYC184-pT7-switch3-LacI	Verifying the orthogonality
T3S4	pUC19-pTet-trigger3-tetR; pACYC184-pT7-switch4-LacI	Verifying the orthogonality
T4S1	pUC19-pTet-trigger4-tetR; pACYC184-pT7-switch1-LacI	Verifying the orthogonality
T4S2	pUC19-pTet-trigger4-tetR; pACYC184-pT7-switch2-LacI	Verifying the orthogonality
T4S3	pUC19-pTet-trigger4-tetR; pACYC184-pT7-switch3-LacI	Verifying the orthogonality
T4S4	pUC19-pTet-trigger4-tetR; pACYC184-pT7-switch4-LacI	Verifying the orthogonality
TOS	pUC19-pTet-trigger(original)-tetR; pACYC184-pT7-switch-LacI	Investigating the function of toehold structure with 5ʹ end of RNA
TNS	pUC19-pTet-trigger(nohairpin)-tetR; pACYC184-pT7-switch-LacI	Investigating the function of toehold structure with 5ʹ end of RNA
T16S	pUC19-pTet-trigger16-tetR; pACYC184-pT7-switch-LacI	Investigating the function of toehold structure with 5ʹ end of RNA
T17S	pUC19-pTet-trigger17-tetR; pACYC184-pT7-switch-LacI	Investigating the function of toehold structure with 5ʹ end of RNA
XOR1	pUC19-pTet-XOR1triggerAB-tetR; pACYC184-pT7-XOR1switch-LacI	Testing the XOR gate
XOR2	pUC19-pTet-XOR2triggerAB-tetR; pACYC184-pT7-XOR2switch-LacI	Testing the XOR gate
NIMP1	pUC19-pTet-NIMP1triggerAB-tetR; pACYC184-pT7-NIMP1switch-LacI	Testing the NIMP gate
NIMP2	pUC19-pTet-NIMP2triggerAB-tetR; pACYC184-pT7-NIMP2switch-LacI	Testing the NIMP gate
IMP1	pUC19-pTet-IMP1triggerAB-tetR; pACYC184-pT7-IMP1switch-LacI	verifying IMP gate
IMP2	pUC19-pTet-IMP2triggerAB-tetR; pACYC184-pT7-IMP2switch-LacI	Testing the IMP gate
AND	pUC19-pTet-ANDinputAB-tetR; pACYC184-pT7-ANDswitch-LacI	Testing the AND gate
OR	pUC19-pTet-ORinputAB-tetR; pACYC184-pT7-ORswitch-LacI	Testing the OR gate

### 2.3 Fluorescence measurement and cross-talk analysis

The constructed strains (Table 2) were cultured using LB medium, and induced at 2 h using 0.1 mol/L IPTG (isopropyl β-D-1-thiogalactopyranoside), 0.25 mg/mL aTc (anhydrotetracycline, SUPERRI) and 0.1 mg/mL HSL (N-(Ketocaproyl)-L-homoserine Lactone). After induction for 8 h, the cells were harvested by centrifugation at 13910 × *g*, and then were washed and resuspended in buffer PBS. Subsequently, the biomass and fluorescence intensity were measured by the plate reader (SYNERGY-HI, Bio-Tek, USA). Briefly, the fluorescence intensity of green fluorescent protein (GFP) was measured at excitation/emission wavelengths of 485 nm/528 nm, and the biomass was measured at 600 nm. Finally, the output (F) was calculated as a follow formula,

(1) $$F=\frac{F-exp}{Abs-exp}-\frac{F-neg}{Abs-neg}$$

where F_exp and Abs_exp represent fluorescence intensity and OD_600_ of experimental groups strains and F_neg and Abs_neg represent fluorescence intensity and OD_600_ of negative control strains. The negative control is the competent BL21 Star DE3 strain without any plasmid. The outputs are identified as ‘0’ and ‘1’ according to the threshold (relative fluorescence intensity: 50%) [4,5].

Cross-talk was determined by dividing the arithmetic mean of the GFP fluorescence intensity from a given trigger switch pair by the arithmetic mean of the GFP fluorescence intensity for the cognate trigger switch interaction.

### 2.4 In silico design and selection of toehold switches

This section describes the initial (computational designing) stages of the toehold switch generation process, including design specification and *in silico* screening and selection.

#### 2.4.1 Design specification: specification of RNA sequences and secondary structures

The first stage in the design process involves the definition of the logic gate secondary structure and interaction domain sizes. The IMP gate RNAs based on toehold switches and 3WJ repressors were generated by taking the core regulatory sequence of the toehold and the 3WJ repressors. There is always a 21-nt linker region between the hairpin module and coding sequence of the regulated gene [14], and the definition of a core regulatory sequence is the sequences running from the 5ʹ end of the binding domain through to the nucleotide immediately before the 21-nt linker sequence. Because the core regulatory of 3WJ repressors sequence has a length of 73-nt, spacers of 3 *n* + 2 in length, where *n* is a non-negative integer, were used to connect different toehold switches and 3WJ repressors. Spacers of this length enabled successive repressor modules to remain in-frame through the full length of the gate RNA. A previous study proved that 17-nt spacers worked well in the NAND gates [14]. So, in this study, a 17-nt spacer was also designed and inserted between the toehold switch hairpin and the 3WJ repressor hairpin in the IMP gate.

The function of the XOR gate is based on its triggers. The trigger RNAs were generated by taking the core sequence of the trigger of the toehold. The core of a trigger is the sequence that can be combined with the switch. The blue-colored regions in Figure 4(b) represent regions with pre-determined sequences, which are the core sequences of the trigger. The red- and purple-colored regions are the complementary domains of the 5ʹ end and the 3ʹ end. The lengths of the complementary domains should be appropriate to ensure the combination, so they were chosen to be 22-nt at each side.

#### 2.4.2 RNA sequence design: NUPACK-based sequence generation

NUPACK design algorithm computes candidate RNA sequences and progressively refines them until their deviation from the specified design constraints falls below a specified stop condition. In designing IMP gate, stop conditions were imposed on switch RNA. In designing the XOR gate, stop conditions were imposed on trigger RNAs. Free energies specified in Serra et al. [19], a temperature of 37°C, 1.0 mol/L Na^+^, and 0.0 mol/L Mg^2+^ were used for the design algorithm. Monomeric repetitive sequence patterns, such as AAAA, CCCC, GGGG, UUUU, KKKKKK, MMMMMM, RRRRRR, SSSSSS, WWWWWW, YYYYYY, were prevented in the design specification to preclude runs of monomeric nucleotides.

IMP design is shown below (using dot-bracket notation):

trials = 10

structure switch = … …. …. ….(((((((((… ((((((…. … ….)))))) …))))))))) …. …. …. …. …. …. … ….((((((… ((((((…. … ….)))))) …)))))) …. …. …. …. ….((….)) … ((((….)))) ….

domain a = GGGAUAAGUAGAUAAGAUUGUUAGAUGGCUUCGAACAGAGGAGACGAAGCAUGCUAACAAUC

domain b = N17

domain c = CUCCUAUCACUUUACUUGUUAUAGUUAUGAACAGAGGAGACAUAACAUGAACAAGCACACUAACUACAAAUUCAACCUGGCGGCAGCGCAAAAGAUGCGUAAA

prevent = aaaa, cccc, gggg, uuuu, kkkkkk, mmmmmm, rrrrrr, ssssss, wwwwww, yyyyyy

switch.seq = a b c

‘domain a’ is a sequence of the toehold, ‘domain b’ is a sequence of spacer and ‘domain c’ is a sequence of the 3WJ repressor.

XOR design is shown below (using dot-bracket notation):

trials = 10

structure trigger1 = … ((((((((… …)))))))) …. …. …. …. …. …. …. …. …. …. …. …. …. …. …. …. …. …. … ….

structure trigger2 = … ((((((((… …)))))))) …. …. …. …. …. …. …. …. …. …. …. …. …. …. …. …. …. …. … ….

domain a = GGG

domain b = N27

domain c = N22

domain d = N22

domain t = core sequence

prevent = aaaa, cccc, gggg, uuuu, kkkkkk, mmmmmm, rrrrrr, ssssss, wwwwww, yyyyyy

trigger1.seq = a b c t d

trigger2.seq = a b d* t c*

‘domain a’ is a GGG leader sequence, ‘domain b’ is a hairpin sequence that minimizes the degradation of triggers, ‘domain c’ and ‘domain d’ are sequences of complementary domains, and ‘domain t’ is the core sequence.

#### 2.4.3 In silico screening: fast removal of unwanted designs

The resulting designs were then screened to ensure they had no duplicate sequences or in-frame stop codons that would prematurely terminate translation of the output gene. The screened designs were then analyzed for their behaviors. We began this process by first computing the pairwise interactions between the trigger RNA and switch RNA sequences. Simulations were performed with NUPACK. The NUPACK functions were run with a specified temperature of 37°C, 1.0 mol/L Na^+^, and 0.0 mol/L Mg^2+^ using Serra et al. [19] free energy parameters and assumed strand concentrations of 1 nM. The output of these functions provided the free energies of the individual RNA strands and the bimolecular trigger-switch complex, and the predicted concentrations and minimum free energy secondary structures of each species in solution. The stimulating sequences that best meet the behaviors requirements and have less Minimum Free Energy (MFE) were selected as the final sequence.

## 3. Result and discussion

The logic gate can integrate multiple input signals following a set of algorithms and generate a response only if strictly defined conditions are met. Therefore, logic gates are the key components for building complex circuits or a complete synthetic biological system. However, the logic gates of IMP and XOR are still not be constructed. To address this, we exploited two types of novel RNA-only biological Boolean logic gates, IMP and XOR, which were built with toehold switch and 3WJ repressor. This section demonstrates that they can achieve the truth tables of logic gates by adding different trigger RNAs. Subsequently, the results of fluorescence analyzing and characterization of their outputs suggested that these logic gates are feasible and prospect for practical use.

### 3.1 Construction and validation of basic parts of toehold and three-way-junction repressor

**Figure 1.**
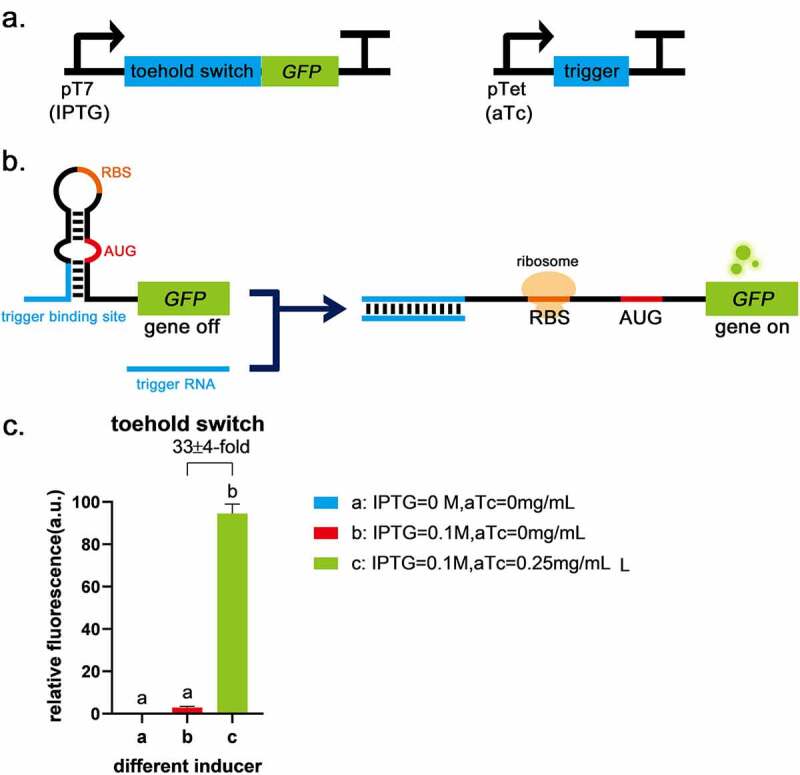
Toehold switch validation a, The circuits of toehold switch and the trigger RNA. b, The schematic of toehold switch. c, Compared with the blank control (IPTG = 0 mol/L, aTc = 0 mg/mL), the fluorescence intensity of GFP was low when only the promoter before toehold sequence was turned on (IPTG = 0.1 mol/L, aTc = 0 mg/mL), indicating that toehold has the advantage of low leakage. When the trigger was expressed (IPTG = 0.1 mol/L, aTc = 0.25 mg/mL), it was shown a significant difference (*P* < 0.01) and up to 32 ± 4-fold induction of GFP fluorescence intensity due to the destruction of the toehold hairpin structure with the second group. Error bar: SD (n = 9).

In a previous study, it was reported that a toehold switch had the characteristics of low leakage and a high ON/OFF ratio [9], so it was chosen as the basic part to construct logic gates. Toehold switch RNA comprises of cis-acting element RNA hairpin and trans-acting factor trigger RNA. The binding of a trigger RNA to the toehold sequence allows for a branch migration process, exposing AUG and RBS for translation initiation (Figure 1(b)). The sequences of toehold were obtained from Green et al. [9] and used to construct plasmids pUC19-pTet-trigger(original)-tetR and pACYC184-pT7-switch-LacI (Table 2, the complete sequences of all parts are given in Supplementary Table S1). The circuit diagram is shown in Figure 1(a). Functional characterization suggests translational repression from the switch RNA and strong active translation upon trigger expression, which provides up to 33-fold GFP enhancement (Figure 1(c)). Compared with the blank control, the fluorescence intensity of GFP was low when the toehold switch was off (Figure 1(c)), indicating that the toehold switch was shown the advantages of high ON/OFF ratio and low leakage. Therefore, the toehold switch can be used for subsequent logic gate construction.

**Figure 2.**
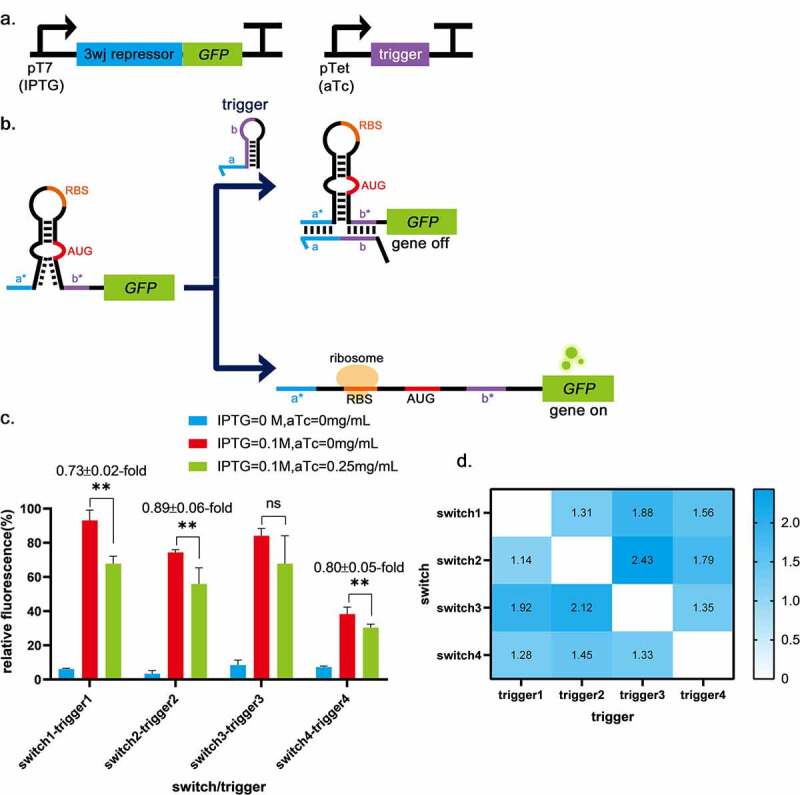
Three-way-junction repressors validation. a, The circuits for the 3WJ repressor and the trigger RNA. b, The schematic of the 3WJ repressor. c, The fluorescence differences between the groups without trigger expression (IPTG = 0.1 mol/L, aTc = 0 mg/mL) and the groups with trigger expression (IPTG = 0.1 mol/L, aTc = 0.25 mg/mL). The groups without switch and trigger expression (IPTG = 0 mol/L, aTc = 0 mg/mL) are control. The fluorescence differences of switch1-trigger1 is 0.73 ± 0.02-fold, switch2-trigger2 is 0.89 ± 0.06-fold and switch4-trigger4 is 0.80 ± 0.05-fold. While switch3-trigger3 was not investigated because the induction of the trigger3 did not significantly decrease the induced fluorescence intensity. ** indicates *P* < 0.01 through One-Way ANOVA analysis. ns: not significant. d, Cross-talk was determined by dividing the arithmetic mean of the GFP fluorescence intensity from a given trigger switch pair by the arithmetic mean of the GFP fluorescence intensity for the cognate trigger switch interaction. GFP fluorescence intensity was measured from n = 9 biologically independent samples.

3WJ repressor switch RNA employs an unstable hairpin secondary structure, which has been demonstrated to be translationally active in the 3WJ repressor [14]. On either side of the unstable hairpin are single-stranded domains. When the trigger RNA is expressed, it binds to the single-stranded domain of the switch RNA. The resulting trigger-switch complex has a stable 3WJ structure that effectively sequesters the RBS and the start codon within the loop and stem of the switch RNA, respectively, and strongly represses translation [14] (Figure 2(b)). Therefore, a 3WJ repressor was chosen as the basic part to construct logic gates. When the input is 0, the output of 3WJ repressor is 1, which works in opposite way to toehold switch. These confer more possibilities for constructing novel RNA-only logic gates. The sequence of 3WJ was obtained from Kim et al. [14] and used to construct plasmid pUC19-pTet-trigger1-tetR, pUC19-pTet-trigger2-tetR, pUC19-pTet-trigger3-tetR, pUC19-pTet-trigger4-tetR, pACYC184-pT7-switch1-LacI, pACYC184-pT7-switch2-LacI, pACYC184-pT7-switch3-LacI and pACYC184-pT7-switch4-LacI (Table 2). The circuit diagram is shown in Figure 2(a). To select the suitable switch-trigger pair, the fluorescence differences between the switch-trigger pair without trigger expression and with trigger expression were evaluated. As shown in Figure 2(c), the fluorescence differences of switch1-trigger1 has the most significant decrease (0.73 ± 0.02-fold) among all the switch-trigger pairs. The dynamic range is significantly lower than the previous reports [14]. It is probably due to the differences in strain culture and fluorescence intensity assay methods.

One of the prerequisites for higher-order logic processing is the orthogonality of regulatory components [14]. Thus, the interactions between pairwise combinations of different repressor trigger and switch RNAs were measured in strains (Table 2), suggesting low levels of cross-talk between the switch and its non-cognate triggers (Figure 2(d)). Considering the significant decrease of the induced fluorescence intensity between the groups without trigger expression and the groups with trigger expression and the orthogonality of switch-trigger pairs, switch1-trigger1 was chosen to build subsequent logic gates.

### 3.2 In silico design of IMPLICATION gate by combining toehold with three-way-junction repressors

**Figure 3.**
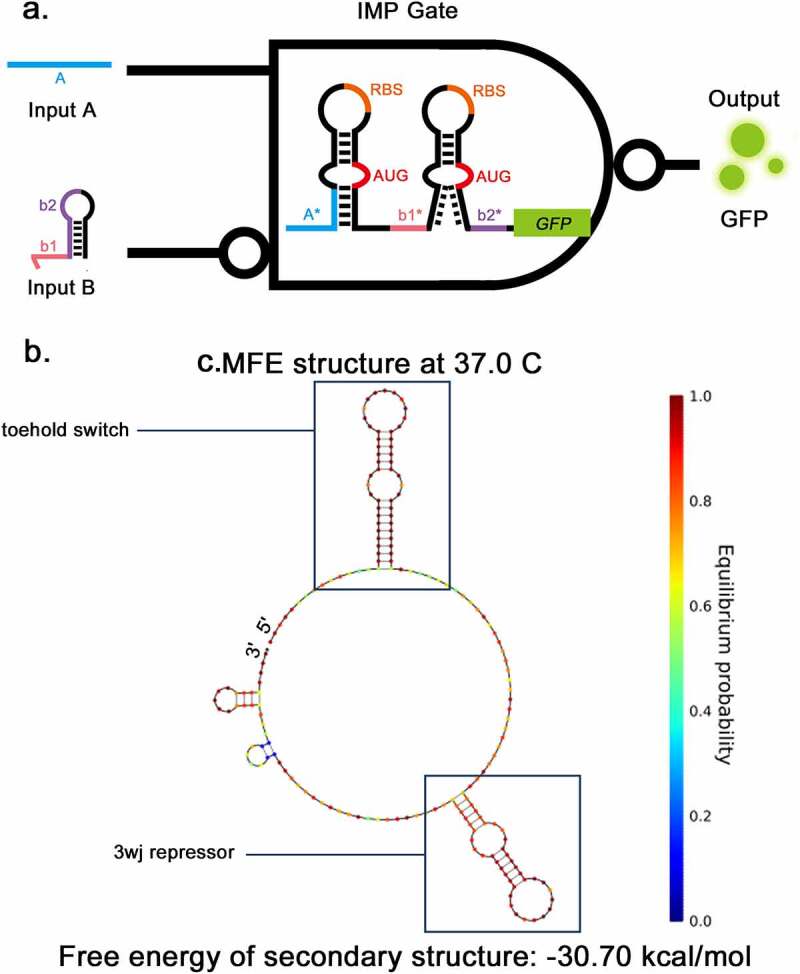
Two-input toehold and three-way-junction repressor IMPLICATION gate a, The structural schematic of IMP gate. The structure of IMP gate consists of a toehold switch and 3WJ repressor, The input A and B are the triggers for the toehold switch and 3WJ repressor respectively. GFP is applied as the output. b, The secondary structure of IMP gate predicted by NUPACK.

Previously, Kim, et al. constructed a NAND gate consisting of two 3WJ repressors [14]. They demonstrated that a ribosome bounded to the RBS can pass through the downstream stable 3WJ hairpin complex. Furthermore, even if the 3WJ hairpin becomes stable after being combined with the trigger RNA, it cannot detach the ribosome from the RNA to stop the translation process. Based on these mechanisms, a new structure combining the toehold switch and 3WJ repressor was designed to implement the IMP Boolean calculation in this study (Figure 3(a)). The sequence was de-novo designed using the NUPACK sequence design package [20] (see Methods for details). The secondary structure of IMP switch-RNA is shown in Figure 3(b), consisting of a toehold switch, 17-nt linker and a 3WJ repressor. The predicted secondary structure of IMP triggers is shown in supplementary Fig S1b-c, the same as the secondary structure of trigger of toehold or 3WJ repressor. The behaviors of the combination of trigger RNA and switch RNA were also predicted by NUPACK (Supplementary Fig S1d). The toehold trigger RNA can cause the corresponding hairpin to unwind and the 3WJ trigger can stabilize the corresponding hairpin. These simulation results suggest that the design of IMP gate is feasible.

The scalability of the ribocomputing devices is also a key indicator of the performance of logic gates [17]. Previously, a 12-input RNA computation was evaluated robustly [17], to enrich the types of logic gates available for implementing complex calculations, IMP gate RNAs based on toehold switch and 3WJ repressors were extended to three-input operation by adding additional toehold switch modules upstream of the logic gate (Supplementary Fig S2). This three-input gate is called a ‘swapping gate’ that can switch between IMP and NOT. Moreover, to enrich the logic gate types available for implementing complex calculations, other possible ‘swapping gates’ that can switch between two kinds of logic gates were also designed (Supplementary Table S1. 6), such as the swapping gate that can achieve the transformation between the AND gate and the OR gate (Supplementary Fig S3). These swapping gates indicate the possible scalability of our novel logic gate.

### 3.3 In silico implement XOR gate by designing complementary domains of triggers at the 5ʹ end and 3ʹ end

**Figure 4.**
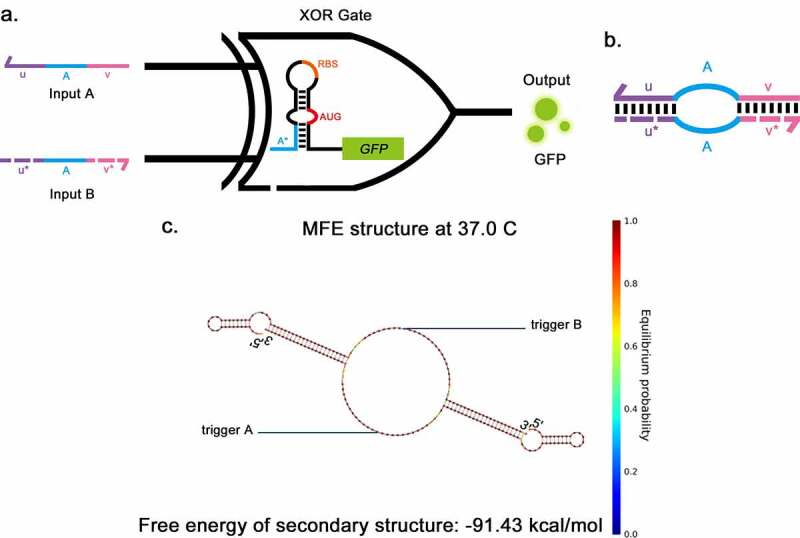
Two-input toehold XOR gate a, The structural schematic of the XOR gate. The structure of the XOR gate consists of a toehold switch and two triggers, The two inputs (a and b) are the triggers for the toehold switch, sharing a common core sequence (a) that allows them to pair with the toehold domain (A*), thus disassembling the stem-loop structure. GFP is applied as the output. b, The structural schematic of the pairing between two input triggers in the XOR gate. The complementary domain of input A (*u, v*) and input B (u*, v*) can pair specifically pair with each other, forming a ring in the middle. c, The secondary structure of triggers of XOR gate when they pair with each other as predicted by NUPACK.

Previously, Green et al. reported an A AND (NOT B) logic gate, consisting of a gate RNA and two regulatory RNAs (trigger RNA and deactivating RNA) [17]. The deactivating RNA can bind directly to trigger RNA to silence its effect on the gate RNA. Taking inspiration from the A AND (NOT B) gate, the XOR gate was developed, consisting of a toehold and two triggers (Figure 4(a)). Two trigger RNAs have the complementary domains at their 5ʹ end and 3ʹ end (u, u*, v and v* in Figure 4(b)). The complementary domain of input A (*u, v*) and input B (u*, v*) can pair specifically with each other, forming a ring in the middle. The sequence was de-novo designed using NUPACK [20] (see Methods for details). The secondary structure of the trigger complex is shown in Figure 4(c), the length of the complementary domain at each side of the triggers was 22-nt, and the core sequences of triggers were derived from previous literature [9,14]. The predicted secondary structure of the XOR switch is shown in supplementary Fig S4a, which is the same as the secondary structure of the toehold switch. The combination of trigger and switch was also predicted by NUPACK (Supplementary Fig S4d). The toehold hairpin can unwind in response to each trigger; meanwhile, the triggers can form a complex when both input triggers are present. This predicted process suggests that using trigger pairs whose ends can be combined to implement XOR function is practicable.

The arithmetic units, such as half adder and subtractor (Supplementary Fig S5), are capable of carrying out simple binary mathematical operations necessary for digital computing. These arithmetic units are often constructed using different types of logic gates. XOR gate is an important part of the half adder [21], thus our research provides possibilities for realizing RNA-only arithmetic units.

### 3.4 In vivo computation using IMPLICATION circuit

**Figure 5.**
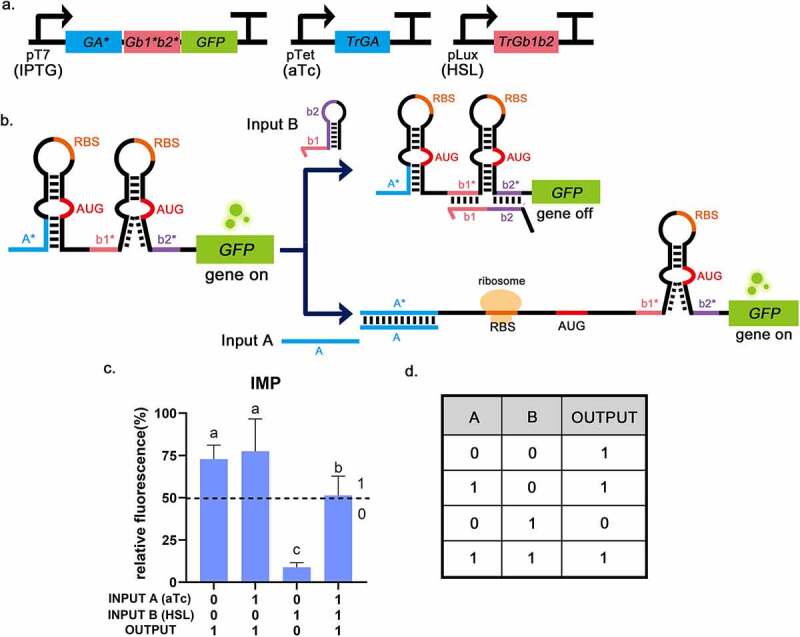
The experimental results of the IMPLICATION gate (B implies A). a, The circuit diagram of IMP gate. b, The operating mechanism of IMP gate. Input A will bind to the toehold switch, allowing for translation initiation. Input B will bind to 3WJ switch RNA to strongly repress translation. c, Relative fluorescence intensity is shown in the picture which the highest fluorescence intensity value is chosen as 100%. It is shown that when INPUT A = 0, INPUT B = 1, the fluorescence intensity of GFP is low. And the fluorescence intensity of GFP was high in the other three groups, corresponding to the situation described in the truth table. INPUT A = 1 means that aTc (0.25 mg/mL) is added, INPUT B = 1 means that HSL (0.1 mg/mL) is added. The dotted lines indicate the corresponding threshold (relative fluorescence intensity: 50%). Error bar: SD (n = 9). *P* < 0.01 through One-Way ANOVA analysis. d, The truth table of the IMP gate (B implies A).

IMP gate, assembled from toehold switch and 3WJ repressor, was supposed to operate in the following way. Trigger RNAs work like the input and GFP as the output. When no trigger RNA was transcribed, IMP gate acted like a 3WJ. Because the RBS and the start codon (AUG) in the unstable hairpin structure were easily exposed, the translation process could occur normally without any trigger RNAs. Input A would bind to the toehold switch, which allowed a branch migration process and exposed RBS and AUG for translation initiation. Input B would bind to the 3WJ switch, and the resulting trigger-switch complex had a stable 3WJ structure that respectively sequestered the RBS and the start codon within the loops of the switch RNA, which strongly repressed the translation. When both input trigger RNAs were added, the RBS of the toehold switch would be exposed and allow ribosome binding, which would break to open the stable 3WJ hairpin, allowing downstream genes to translate (Figure 5(a,b)). The output was divided into 0 and 1 according to the threshold (relative fluorescence intensity: 50%) [4,5]. Measurements of IMP gate was shown a high GFP fluorescence intensity in the logical TRUE condition (Output = 1), which was at least six times higher than the logical FALSE condition (Output = 0) that only trigger-RNA B was inputted (Figure 5(c,d)). These results indicate that the connection of toehold switch and 3WJ repressor can implement the IMP function. In previous studies, all logic gates were all assembled using the same type of logic gates, and this form could only implement different logical operations by changing the number of unit structures of RNA switches, which had certain limitations [17]. In this study, the IMP gate provided the first structural assembly of the toehold switch and 3WJ repressor, meaning that multiple types of RNA switches can be used in different combinations, bringing the possibility for constructing complex logic gates.

### 3.5 In vivo computation using XOR circuit

**Figure 6.**
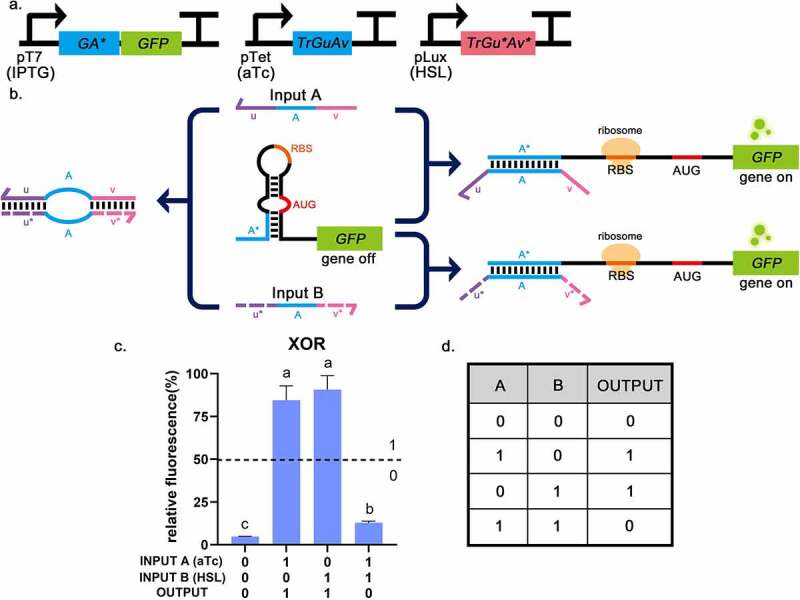
The experimental results of the XOR gate. a, The circuit diagram of the XOR gate. b, The operating mechanism of the XOR gate. The toehold switch can be turned ON when either of the triggers is input. When both trigger RNAs are input simultaneously, they will pair with each other and form a ring in the middle, so that the switch will remain in the OFF state. c, Relative fluorescence intensity is shown in the picture which the highest fluorescence intensity value is chosen as 100%. It is shown that when INPUT A and INPUT B are both 0 (or 1), the fluorescence intensity of GFP is low. And the fluorescence intensity of GFP was high in the other two groups. This corresponds to the situation described in the truth table. INPUT A = 1 means that aTc (0.25 mg/mL) is added, INPUT B = 1 means that HSL (0.1 mg/mL) is added. The dotted lines indicate the corresponding threshold (relative fluorescence intensity: 50%). Error bar: SD (n = 9). *P* < 0.01 through One-Way ANOVA analysis. d, The truth table of the XOR gate.

For the XOR gate, the key to ensuring that the triggers work as expected is that the 5ʹ end and the 3ʹ end complementary domains bind to each other through desired base pairing. If they could combine successfully without affecting the functionality of the intermediate sequence, the logic gate works. The toehold switch had a stable hairpin structure and concealed RBS and AUG in the loop region, so when there was no input trigger, the ribosome would not find the binding site, and the translation process would not start. Since both triggers contained sequences that can open the hairpin, the toehold switch could be turned ON when either of them is input. However, when both triggers were input simultaneously, they pair with each other and form a ring in the middle, so the switch remains in the OFF state (Figure 6(a,b)). The outputs which were larger than the threshold value (relative fluorescence intensity: 50%) were in the ‘0’ state and others were in the ‘1’ state [4,5]. GFP fluorescence intensity was significantly reduced when no input or both inputs are present. The best TRUE (Output = 1) state had a 19-fold increase in GFP expression compared to the logical FALSE states (Output = 0). Only a single input could produce a TRUE state (Output = 1), which conformed to the truth table of the XOR gate (Figure 6(c,d)). This indicates that the construction of XOR gate is successful and the design of complementary binding domains at both ends of the trigger does not affect the function of the intermediate. The special design of triggers is not just for building the XOR gate but can be useful in many practical scenarios. For example, triggers can be designed to regulate specific microRNAs (miRNAs). In this way, complex regulation can be implemented, so that the regulatory process is not limited to a simple promotion or inhibition, and can work in complex gene regulation networks.

### 3.6 The development and application prospect of boolean logic gate

In recent years, molecular platforms based on logic gates are maturing, and all kinds of mainstream logic gates and cascade logic gates have been constructed one by one [22]. In principle, the logic gates presented in this study can even utilize trans-acting factors, such as the σ factor, to achieve a cascade of logic gates consisting of RNA elements [23]. However, these ideas are still in the early designing stage and need further investigation. In addition, we proposed a new conceptual ‘swapping gate’ that can switch from one type of logic gate to a different type, providing the possibilities for developing novel RNA circuits. Two ‘swapping gates’ were designed which can switch between the two different types of logic gates. The first Swapping gate was constructed from the inhibitory hairpin and the 3WJ repressor, which achieved the transformation between IMP and NOT. If trigger A was input, it was an IMP gate. If not, it was a NOT gate (Supplementary Fig S2). The second swapping gate combines the single hairpin switch and the inhibitory hairpin switch, which could switch from the AND gate to the OR gate. When trigger B was input, it was an OR gate. When trigger A was absent, it behaved like an AND gate (Supplementary Fig S3).

Boolean logic gates have a wide range of applications, for example, in the construction of arithmetic units, including the half adder and the subtractor [21,24–27]. The arithmetic units are capable of carrying out simple binary mathematical operations necessary for digital computing (Supplementary Fig S5).

Moreover, these logic gates may have many practical applications, such as virus detection and disease treatment. The programmability of RNA-based regulatory elements is based on the complementary sequence pairing, which guarantees a sufficient specificity for biosensors. By designing specific regulatory elements, multiple viruses could be detected simultaneously in an orthogonal test. By combining different types of logic gates, the performance of biosensors can be improved by avoiding false negatives and false positives. For example, an OR gate can reduce false negatives, an AND gate can reduce false positives, and an IMP gate can exclude non-homologous viruses using a highly conserved viral sequence as the input (Figure 5(b), trigger B). According to the expression of different logic-gate reporters, we can determine the presence of multiple viruses (Supplementary Fig S6). Concerning disease treatment, microRNA and long non-coding RNA play crucial roles in various diseases, including cardiovascular diseases, hepatitis, diabetes mellitus, cancer, etc [28–32]. If miRNAs are used as inputs to these logic gates, it may provide an alternative way of thinking about disease diagnosis and treatment [33,34]. If these ideas become reality, biological logic gates could be used to accomplish special tasks in many aspects of lives of people.

## 4. Conclusion

In this study, the IMP gate based on the toehold switch and the 3WJ repressor and the XOR gate based on trigger pairs with complementary ends, were constructed successfully. Furthermore, the IMP gate provided the first structural assembly of the toehold switch and 3WJ repressor, suggesting that multiple types of RNA switches can be used in combination. For the XOR gate, the 5ʹ end and the 3ʹ end complementary domains of trigger RNAs could combine successfully without affecting the functionality of the intermediate sequence. The novel RNA-only IMP and XOR logic gates have wide dynamic ranges, less cross-talk and logical computing power. These advantages confer their possibility of designing novel biological circuits and broadening their applications. It is still worth exploring how to give the normalized parameters of fluorescence intensity to make the logic gate universal.

## Supplementary Material

Supplemental Material

## Credit author statement

Chaoxin Chen: Project administration, Methodology, Writing - original draft. Qi Wu: Project administration, Software, Writing - original draft. Qingying Ke: Investigation, Visualization, Writing - original draft. Ting Wang: Data curation, Methodology. Yifan Zhang: Data curation, Methodology. Feiwen Wei: Data curation. Xiaolong Wang: Supervision, Writing - review & editing. Guanglei Liu: Conceptualization, Supervision, Writing - review & editing. All authors read and approved the manuscript.
