# Supplementary material for: Implementation of novel boolean logic gates for IMPLICATION and XOR functions using riboregulators
Source: Bioengineered. 2022 Jan 5;13(1):1235–48. doi: 10.1080/21655979.2021.2020493 (PMC8805959; doi:10.1080/21655979.2021.2020493)
Supplement: Supplemental Material [file KBIE_A_2020493_SM1369.zip › supplementary/downloadFromZipFile.pdf]

Supplementary information

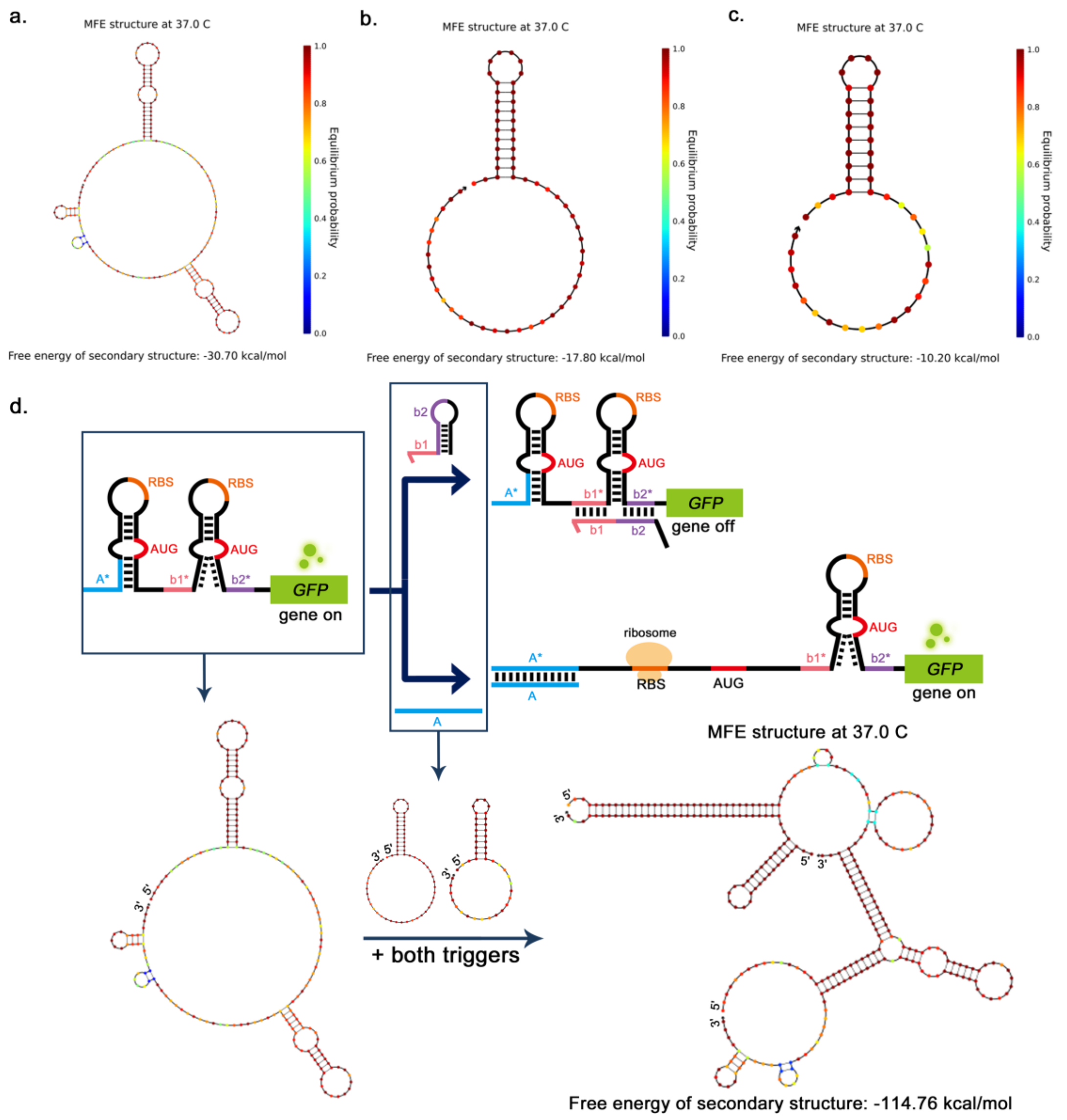

Supplementary Figure S1. IMP gate. a, The secondary structure of IMP switch predicted by NUPACK, free energy of secondary structure: -30.70 kcal/mol. b, The secondary structure of IMP trigger predicted by NUPACK, free energy of secondary structure: -17.80 kcal/mol. c, The secondary structure of IMP trigger predicted by NUPACK, free energy of secondary structure: -10.20 kcal/mol. d, The operating mechanism and the corresponding secondary structure of the IMP gate.

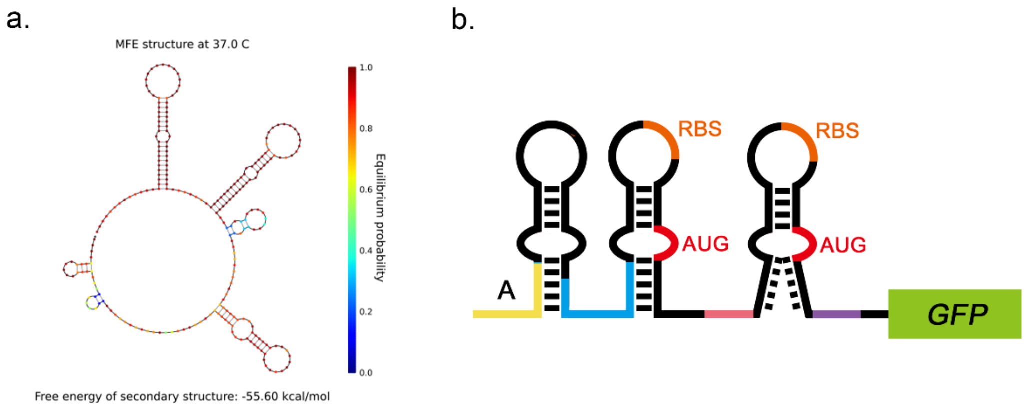

Supplementary Figure S2. The triggers used to control the transformation between two logic gates. a, The triggers used to control the transformation between two logic gates. The inhibitory hairpin constructed switch was combined with the 3WJ repressor, which achieved the transformation between IMP and NOT. If trigger A is input, it is an IMP gate. If trigger A is not input, the inhibitory hairpin will prevent the second hairpin, and this swapping gate is just like a single 3WJ repressor, which can work as a NOT gate. b, One of the secondary structure of IMP and NOT swapping gate predicted by NUPACK, free energy of secondary structure: -55.60 kcal/mol.

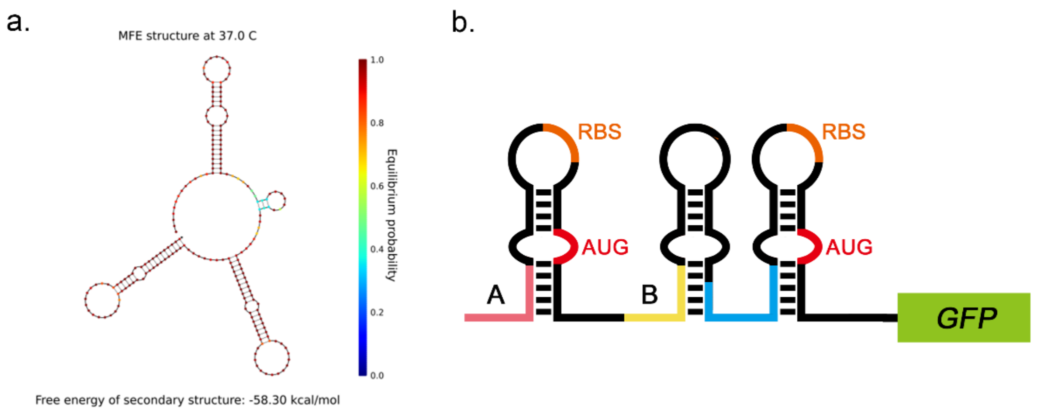

Supplementary Figure S3. Triggers used to control the transformation. a, Triggers used to control the transformation. Combining the single hairpin constructed switch and the inhibitory hairpin constructed switch can achieve the transformation between AND gate and OR gate. Trigger A is for toehold switch and trigger B is for the inhibitory hairpin respectively. When trigger A is absent, this gate is like the inhibitory hairpin constructed switch. And when trigger B is input, this gate is like consisting of two toehold switch and can work as OR gate. b, One of the secondary structure of AND and OR swapping gate predicted by NUPACK, free energy of secondary structure: -58.30 kcal/mol.

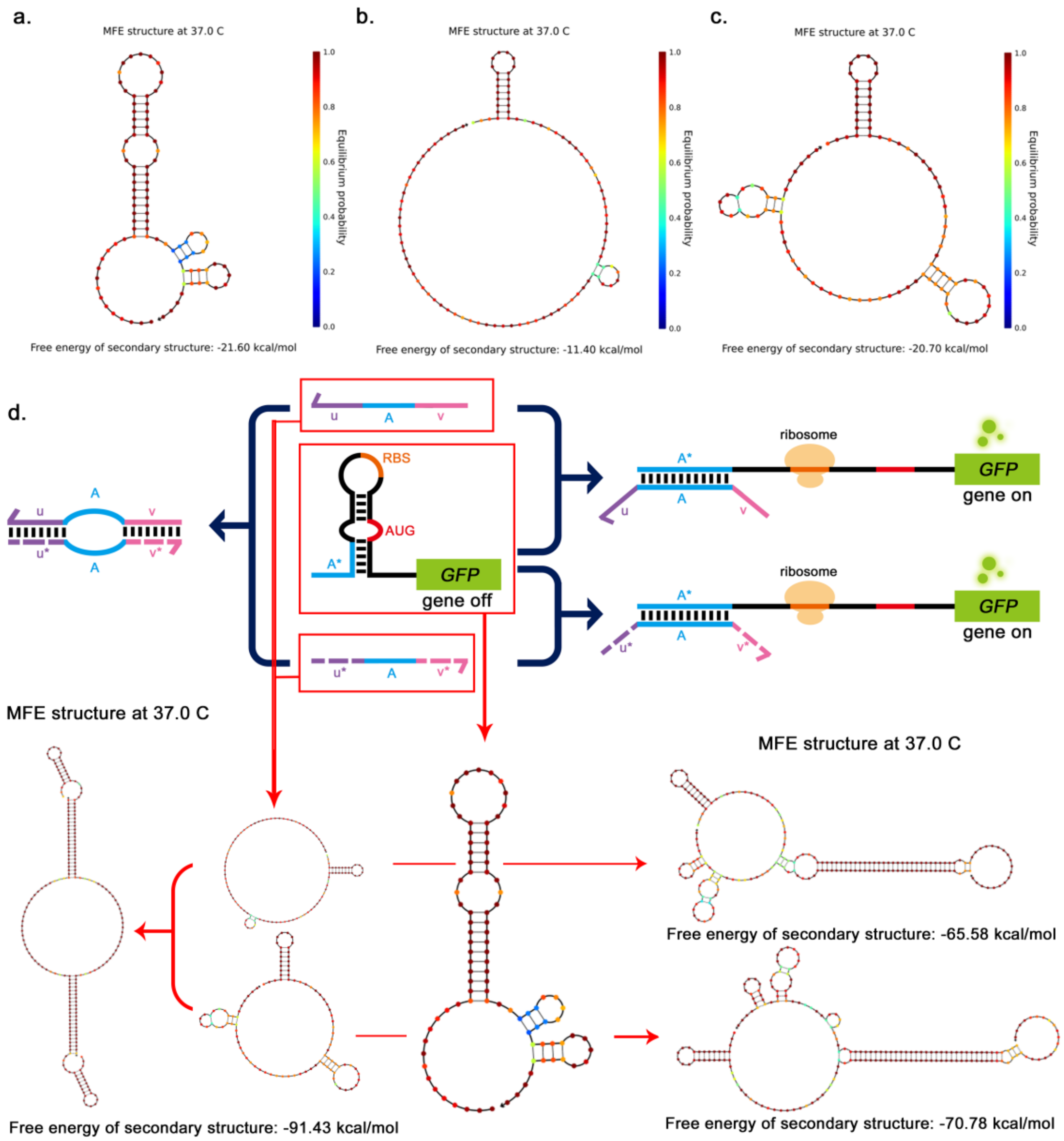

Supplementary Figure S4. XOR gate. a, The secondary structure of XOR switch predicted by NUPACK, free energy of secondary structure: -21.60 kcal/mol. b, The secondary structure of XOR trigger predicted by NUPACK, free energy of secondary structure: -11.40 kcal/mol. c, The secondary structure of XOR trigger predicted by NUPACK, free energy of secondary structure: -20.70 kcal/mol. d, The operating mechanism and the corresponding secondary structure of the XOR gate.

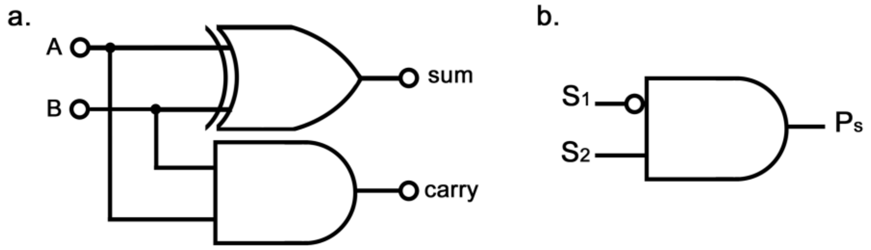

Supplementary Figure S5. The half-adder and the subtractor. a. The half-adder consists of an AND gate and an XOR gate. A half adder is a type of adder, an electronic circuit that performs the addition of numbers. The half adder can add two single binary digits and provide the output plus a carrying value. It has two inputs, A and B, and two outputs S (sum) and C (carry). b. The subtractor consists of a NIMP gate. The subtractor can be done by adjusting the concentration of trigger RNAs. When two triggers are expressed simultaneously, trigger S1 and trigger S2 will combine to form a double-stranded RNA, which cannot be turned on by RNA Switch. Only when trigger S2 is expressed, the downstream gene expression of RNA Switch will be turned on. This subtracter can simulate S2-S1=Ps, in which Ps is the fluorescence intensity of the reporter gene.

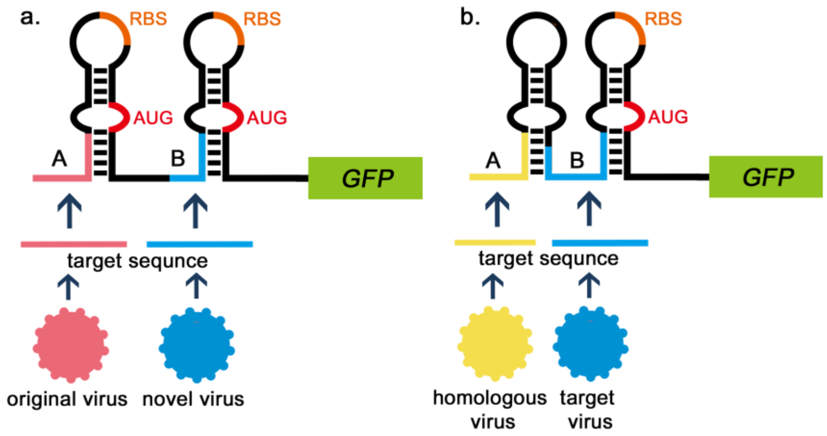

Supplementary Figure S6. Detection of virus. a, A false negative is a major challenge in virus testing. viruses often produce mutated individuals, testing for a single fragment may cause the ignorance of potentially dangerous viruses. Using OR gate can effectively solve this problem, we can select two target segments, one specific for the original virus and the other for the novel virus. When either of these two targets is present, the reporter gene is expressed. b, False positives are another major challenge in virus testing. Many homologous viruses have numerous highly similar or even identical conserved fragments. Using the AND gate containing an inhibiting hairpin, the specificity of the detection was increased significantly. We designed the binding domain to inhibit the hairpin based on the same sequence of the homologous virus and the second hairpin based on the specific fragment of the target virus.

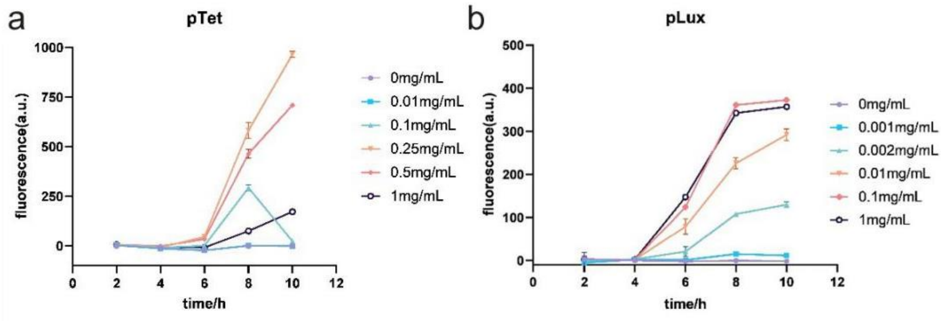

Supplementary Figure S7. The fluorescence intensity chart of promoters with the optimal concentration of inducers as inputs to the logic gates. a, The Tet promoter fluorescence intensity in *E. coli* BL21 (DE3) at different aTc concentrations (0mg/mL, 0.01mg/mL, 0.1mg/mL, 0.25mg/mL, 0.5mg/mL and 1mg/mL). The Tet promoter showed the highest fluorescence intensity when the aTc concentration was 0.25mg/mL. b, The Lux promoter fluorescence intensity in *E. coli* BL21 (DE3) at different HSL (N-(Ketocaproyl)-L-homoserine Lactone) concentrations (0mg/mL, 0.001mg/mL, 0.002mg/mL, 0.01mg/mL, 0.1mg/mL and 1mg/mL). The Lux promoter showed the highest fluorescence intensity when the HSL concentration was 0.01mg/mL. Error bar: SD (n=9). Each GFP fluorescence value is normalized to the number of cells by dividing by the OD600. The fluorescence was reported by calculating F= [(Fexperimental/Absexperimental) – (Fnegative control/Absnegative control)], where the negative control was the competent BL21 (DE3) strain without a plasmid.

Supplementary Table S1. Sequences

1.reporter				
Part number	sequences	part library link	describe	curcuit diagram
BBa_K3328031	atgcgtaaaggagaagaacttttcactggagttgtcccaattcttgttgaattagatggtgatgttaatgggcacaaattttctgtcagtggagagggtgaaggtgatgcaacatacggaaaacttacccttaaatttatttgcactactggaaaactacctgttccgtggccaacacttgtcactactttcggttatggtgttcaatgctttgcgagatacccagatcacatgaaacagcatgactttttcaagagtgccatgcccgaaggttacgtacaggaaagaactatatttttcaaagatgacgggaactacaagacacgtgctgaagtcaagtttgaaggtgatacccttgttaatagaatcgagttaaaaggtattgattttaaagaagatggaaacattcttggacacaaattggaatacaactataactcacacaatgtatacatcatggcagacaaacaaaagaatggaatcaaagttaacttcaaaattagacacaacattgaagatggaagcgttcaactagcagaccattatcaacaaaatactccgattggcgatggccctgtccttttaccagacaaccattacctgtccacacaatctgccctttcgaaagatcccaacgaaaagagagaccacatggtccttcttgagtttgtaaccgctgctgggattacacatggcatggatgaactatacaaaaggcctgccgcaaacgacgaaaactacgctgcatcagtttaa	http://parts.igem.org/wiki/index.php?title=Part:BBa_K3328031	GFPmut3b-ASV	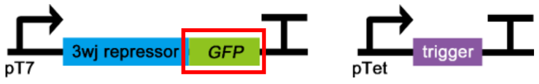
**2.promoter**				
**Part number**	**sequences**	**part library link**	**describe**	**curcuit diagram**
Part:BBa_K2904111	ctcgagaaatcataaaaaatttatttgcttccctatcagtgatagagtataatagagtcgaattgttagcggagaagaatttcacacatcaac	http://parts.igem.org/wiki/index.php?title=Part:BBa_K2904111	Tet_promoter	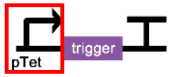
BBa_R0062	acctgtaggatcgtacaggtttacgcaagaaaatggtttgttatagtcgaataaa	http://parts.igem.org/wiki/index.php?title=Part:BBa_R0062	Lux_promoter	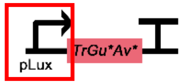

3.sequences for the basic part——3WJ and toehold

Part number	sequences	part library link	describe	curcuit diagram
BBa_K3328000	atgaatgatatacacttgttatagttatgaacagaggagacataacatgaacaagcacgaattgactacactaaacctggcggcagcgcaaaag	http://parts.igem.org/wiki/index.php?title=Part:BBa_K3328000	3WJ_repressor_1	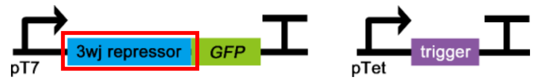
BBa_K3328001	actaatcagatctacttgttatagttatgaacagaggagacataacatgaacaagcacctaacaagactaatcaacctggcggcagcgcaaaag	http://parts.igem.org/wiki/index.php?title=Part:BBa_K3328001	3WJ_repressor_2	
BBa_K3328002	tagtaagattagatattgttatagttatgaacagaggagacataacatgaacaagtacaagaaccgaaacgagaacctggcggcagcgcaaaag	http://parts.igem.org/wiki/index.php?title=Part:BBa_K3328002	3WJ_repressor_3	
BBa_K3328003	ctcctatcactttacttgttatagttatgaacagaggagacataacatgaacaagcacactaactacaaattcaacctggcggcagcgcaaaag	http://parts.igem.org/wiki/index.php?title=Part:BBa_K3328003	3WJ_repressor_4	
BBa_K3328004	gggacgaattgatttgtcaattcgtgcgtgtatatcattcatcat	http://parts.igem.org/wiki/index.php?title=Part:BBa_K3328004	Trigger_of_3WJ_repressor_1	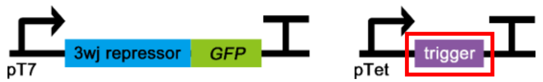
BBa_K3328005	gggacctaacataaacttgttaggtgcgtagatctgattagtgtg	http://parts.igem.org/wiki/index.php?title=Part:BBa_K3328005	Trigger_of_3WJ_repressor_2	
BBa_K3328006	gggacaagaacaatacggttcttgtactatctaatcttactaaac	http://parts.igem.org/wiki/index.php?title=Part:BBa_K3328006	Trigger_of_3WJ_repressor_3	
BBa_K3328007	gggacactaaccatatagttagtgtgcgtaaagtgataggagtaa	http://parts.igem.org/wiki/index.php?title=Part:BBa_K3328007	Trigger_of_3WJ_repressor_4	
BBa_K3328008	gggtagatattgaatgctgctgttatgtcgttaaacagaggagataacgaatgaacagcagcaacctggcggcagcgcaaaag	http://parts.igem.org/wiki/index.php?title=Part:BBa_K3328008	Toehold-switch	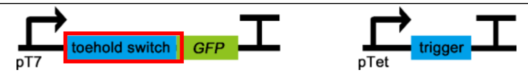
BBa_K3328009	acgtcgactctcgagtgagattgttgacggtaccgtatttttaacgacataacagcagcattcaatatcta	http://parts.igem.org/wiki/index.php?title=Part:BBa_K3328009	Trigger_of_toehold_switch	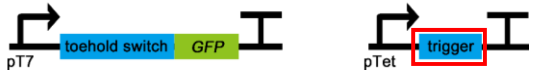
BBa_K3328010	gggtagatattgaatgctgctgttatgtcgttaaacagaggagataacgaatgaacagcagcaacctggcggcagcgcaaaag	http://parts.igem.org/wiki/index.php?title=Part:BBa_K3328010	Trigger_of_toehold_switch	
BBa_K3328011	acgtcgacttatctcgagactgcagttcaatagagatattgttgacggtaccgtatttttaacgacataacagcagcattcaatatcta	http://parts.igem.org/wiki/index.php?title=Part:BBa_K3328011	Trigger_of_toehold_switch	
BBa_K3328012	gggacgcgaaatgctattccatttcgcgtgactaacgacataacagcagcattcaatatctaaac	http://parts.igem.org/wiki/index.php?title=Part:BBa_K3328012	Trigger_of_toehold_switch	

4.sequences for the XOR gate				
**Part number**	**sequences**	**part library link**	**describe**	**curcuit diagram**
BBa_K3328032	ggaattgatattgtagtatgttgaagtgattgaacagaggagacaatcaatgcaacatact aacctggcggcagcgcaaaag	http://parts.igem.org/wiki/index.php?title=Part:BBa_K3328032	Switch_of_XOR_gate_1	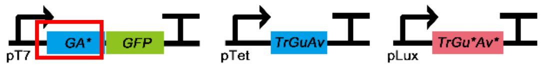
BBa_K3328033	gggcctgaccagttgtatggtcagggtacaagaatagaagcgaagtaaaggtcaatcac ttcaacatactacaatatcaattgctcgggaatagtgcataaagt	http://parts.igem.org/wiki/index.php?title=Part:BBa_K3328033	TriggerA_of_XOR_gate_1	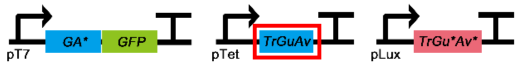
BBa_K3328034	gggtcgttatcgttacagataacgaactacactttatgcactattcccgagccaatcacttca acatactacaatatcaattacctttacttcgcttctattct	http://parts.igem.org/wiki/index.php?title=Part:BBa_K3328034	TriggerB_of_XOR_gate_1	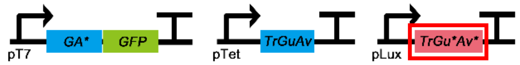
BBa_K3328035	ggtataagtaaatcgcttgctgtatgtcgttaaacagaggagataacgaatgacagcaag caacctggcggcagcgcaaaag	http://parts.igem.org/wiki/index.php?title=Part:BBa_K3328035	Switch_of_XOR_gate_2	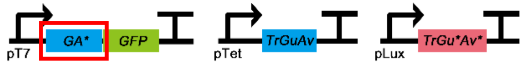
BBa_K3328036	gggcgacagggtcaggtccctgtcgataagatggataggagtaatgaataggtaacga catacagcaagcgatttacttatagggatggagttggaataggagt	http://parts.igem.org/wiki/index.php?title=Part:BBa_K3328036	TriggerA_of_XOR_gate_2	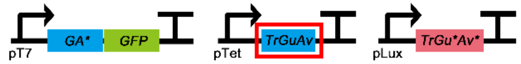
BBa_K3328037	gggcgacagggtcaggtccctgtcgataagactcctattccaactccatccctaacgacat acagcaagcgatttacttatacctattcattactcctatccat	http://parts.igem.org/wiki/index.php?title=Part:BBa_K3328037	TriggerB_of_XOR_gate_2	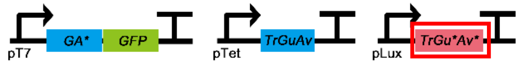

5.sequences for the IMP gate				
Part number	sequences	part library link	describe	curcuit diagram
BBa_K3328038	ggaattgatattgtagtatgttgaagtgattgaacagaggagacaatcaatgcaacatactaaacagataaagcgacgatgaatgatatacacttgttatagttatgaacagaggagacataacatgaacaagcacgaattgactacactaaacctggcggcagcgcaaaag	http://parts.igem.org/wiki/index.php?title=Part:BBa_K3328038	Switch_of_IMP_gate_1	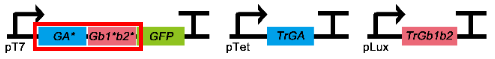
BBa_K3328039	gggacatacggactcacgtgtccgtatgtcagcaatcacttcaacatactacaatatcaattact	http://parts.igem.org/wiki/index.php?title=Part:BBa_K3328039	TriggerA_of_IMP_gate_1	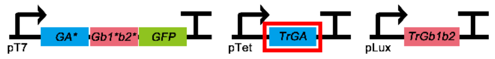
BBa_K3328004	gggacgaattgatttgtcaattcgtgcgtgtatatcattcatcat	http://parts.igem.org/wiki/index.php?title=Part:BBa_K3328004	TriggerB_of_IMP_gate_1	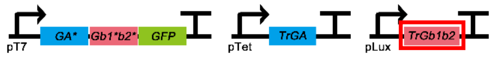
BBa_K3328041	ggaattgatattgtgattatgtgatgattgtaaacagaggagatacaatatgcacataatcactaaagacacagaagaatgaatgatatacacttgttatagttatgaacagaggagacataacatgaacaagcacgaattgactacactaaacctggcggcagcgcaaaag	http://parts.igem.org/wiki/index.php?title=Part:BBa_K3328041	Switch_of_IMP_gate_2	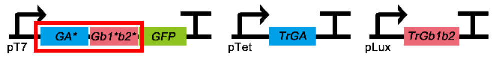
BBa_K3328042	gggacatacggactcacgtgtccgtatgtcaatacaatcatcacataatcacaatatcaattact	http://parts.igem.org/wiki/index.php?title=Part:BBa_K3328042	TriggerA_of_IMP_gate_2	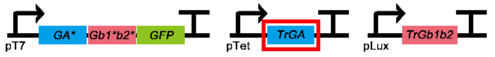
BBa_K3328004	gggacgaattgatttgtcaattcgtgcgtgtatatcattcatcat	http://parts.igem.org/wiki/index.php?title=Part:BBa_K3328004	TriggerB_of_IMP_gate_2	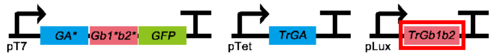

6.sequences for the swapping gate				
Part number	sequences	part library link	describe	curcuit diagram
1	gggcuuaaucuuaccuucgcuuguucuguuccgauacagaaacagaggagauaucggaugagaacaagcacuugcaagcaccuaauuauguaauugauuuggcuucuguuaguuucauacaagaacuuagacaauaugaaaucaacagaagcuacuacuuaccauugucuugcucuauacagaaacagaggagauauagaaugagacaaugg	No upload	Swapping_gate_OR+AND	
2	gggaauugauauuguaguauguugaagugauugaacagaggagacaaucaaugcaacauacuaaacagauaaagcgacguauguaauugauuuggcuucuguuaguuucauacaagaacuuagacaauaugaaaucaacagaagcuacuacuuaccauugucuugcucuauacagaaacagaggagauauagaaugagacaaugg	No upload	Swapping_gate_OR+AND	
3	gggaauugauauugugauuaugugaugauuguaaacagaggagauacaauaugcacauaaucacuaaagacacagaagauauguaauugauuuggcuucuguuaguuucauacaagaacuuagacaauaugaaaucaacagaagcuacuacuuaccauugucuugcucuauacagaaacagaggagauauagaaugagacaaugg	No upload	Swapping_gate_OR+AND	
4	uauguaauugauuuggcuucuguuaguuucauacaagaacuuagacaauaugaaaucaacagaagcuacuacuuaccauugucuugcucuauacagaaacagaggagauauagaaugagacaauggaaacagauaaagcgacgacuaaucagaucuacuuguuauaguuaugaacagaggagacauaacaugaacaagcaccuaacaagacuaaucaaccuggcggcagcgcaaaagaugcguaaa	No upload	Swapping_gate_IMP+AND	
5	uauguaauugauuuggcuucuguuaguuucauacaagaacuuagacaauaugaaaucaacagaagcuacuacuuaccauugucuugcucuauacagaaacagaggagauauagaaugagacaauggaaacagauaaagcgacggggcuccuaucacuuuacuuguuauaguuaugaacagaggagacauaacaugaacaagcacacuaacuacaaauucaaccuggcggcagcgcaaaagaugcguaaa	No upload	Swapping_gate_IMP+AND	
6	uauguaauugauuuggcuucuguuaguuucauacaagaacuuagacaauaugaaaucaacagaagcuacuacuuaccauugucuugcucuauacagaaacagaggagauauagaaugagacaauggacuaaagacacagaagaaugaaugauauacacuuguuauaguuaugaacagaggagacauaacaugaacaagcacgaauugacuacacuaaaccuggcggcagcgcaaaagaugcguaaa	No upload	Swapping_gate_IMP+AND	
7	uauguaauugauuuggcuucuguuaguuucauacaagaacuuagacaauaugaaaucaacagaagcuacuacuuaccauugucuugcucuauacagaaacagaggagauauagaaugagacaauggacuaaagacacagaagaacuaaucagaucuacuuguuauaguuaugaacagaggagacauaacaugaacaagcaccuaacaagacuaaucaaccuggcggcagcgcaaaagaugcguaaa	No upload	Swapping_gate_IMP+AND	
8	uauguaauugauuuggcuucuguuaguuucauacaagaacuuagacaauaugaaaucaacagaagcuacuacuuaccauugucuugcucuauacagaaacagaggagauauagaaugagacaauggacuaaagacacagaagauaguaagauuagauauuguuauaguuaugaacagaggagacauaacaugaacaaguacaagaaccgaaacgagaaccuggcggcagcgcaaaagaugcguaaa	No upload	Swapping_gate_IMP+AND	
9	uauguaauugauuuggcuucuguuaguuucauacaagaacuuagacaauaugaaaucaacagaagcuacuacuuaccauugucuugcucuauacagaaacagaggagauauagaaugagacaauggacuaaagacacagaagacuccuaucacuuuacuuguuauaguuaugaacagaggagacauaacaugaacaagcacacuaacuacaaauucaaccuggcggcagcgcaaaagaugcguaaa	No upload	Swapping_gate_IMP+AND	

10	gggucuuaucuuaucuaucucguuuaucccugcauacagaaacagaggagauaug<br>caaugauaaacgagaaacagauaaagcgacgacaaucaaauacaaauuguuauag<br>uuaugaacagaggagacauaacaugaacaaucacauacaaagcaaacgacaaauac<br>aaauacuaaucagaucuacuuguuauaguuaugaacagaggagacauaacaugaa<br>caagcaccuaacaagacuaaucaaccuggcggcagcgcaaaagaugcguaaa	No upload	Swapping_gate_IMP+NAND	
11	gggaucuauuacuacuuaccauugucuugcucuauacagaaacagaggagauaua<br>gaaugagacaauggaaacagauaaagcgacgacaaucaaauacaaauuguuauag<br>uuaugaacagaggagacauaacaugaacaaucacauacaaagcaaacgacaaauac<br>aaauacuaaucagaucuacuuguuauaguuaugaacagaggagacauaacaugaa<br>caagcaccuaacaagacuaaucaaccuggcggcagcgcaaaagaugcguaaa	No upload	Swapping_gate_IMP+NAND	
12	gggcgauuauggauuagagcuccguuuacugucauacaagaacagaggagauau<br>gacaugaaacggagcaaacagauaaagcgacgacaaucaaauacaaauuguuaua<br>guuaugaacagaggagacauaacaugaacaaucacauacaaagcaaacgacaaau<br>acaaauacuaaucagaucuacuuguuauaguuaugaacagaggagacauaacaug<br>aacaagcaccuaacaagacuaaucaaccuggcggcagcgcaaaagaugcguaaa	No upload	Swapping_gate_IMP+NAND	
13	gggucuuaucuuaucuaucucguuuaucccugcauacagaaacagaggagauaug<br>caaugauaaacgagacuaaagacacagaagaaauugauauuguaguauguugaag<br>ugauugaacagaggagacaaucaaugcaacauacuaaacagauaaagcgacgaug<br>aaugauauacacuuguuauaguuaugaacagaggagacauaacaugaacaagcac<br>gaauugacuacacuaaaccuggcggcagcgcaaaagaugcguaaa	No upload	Swapping_gate_IMP+OR	
14	gggucuuaucuuaucuaucucguuuaucccugcauacagaaacagaggagauaug<br>caaugauaaacgagaaacagauaaagcgacgaauugauauugugauuaugugau<br>gauuguaaacagaggagauacaauaugcacauaaucacuaaagacacagaagaau<br>gaaugauauacacuuguuauaguuaugaacagaggagacauaacaugaacaagca<br>cgaauugacuacacuaaaccuggcggcagcgcaaaagaugcguaaa	No upload	Swapping_gate_IMP+OR	
